# The Role of Continuous Positive Airway Pressure (CPAP) Therapy in Obstructive Sleep Apnea With Metabolic Syndrome: Does It Affect the Outcomes?

**DOI:** 10.7759/cureus.57807

**Published:** 2024-04-08

**Authors:** Sumer S Choudhary, Chetan R Khedkar, Gaurang M Aurangabadkar, Shafee M Khan, Jaydeep G Nayse

**Affiliations:** 1 Respiratory Medicine, Datta Meghe Medical College, Datta Meghe Institute of Higher Education & Research, Nagpur, IND; 2 General Medicine, King Edward Memorial Hospital and Seth Gordhandas Sunderdas Medical College, Mumbai, IND; 3 Community Medicine, Narendra Kumar Prasadrao (NKP) Salve Institute of Medical Sciences & Research Centre and Lata Mangeshkar Hospital, Nagpur, IND

**Keywords:** dyslipidemia, obstructive sleep apnoea, systemic hypertension, obesity and metabolic syndrome, continuous positive airway pressure (cpap)

## Abstract

Background

Of all fatalities occurring globally each year caused by noncommunicable diseases, obstructive sleep apnea (OSA) and obesity are associated with an increased risk of sudden death and cardiovascular mortality. Metabolic syndrome and its comorbidities are linked to OSA. The three essential elements of the metabolic syndrome are improper lipid metabolism, hypertension, and insulin resistance. The effect of continuous positive airway pressure (CPAP) on metabolic syndrome elements and related symptoms and whether CPAP therapy helps reverse the syndrome was studied.

Methods

The present study is prospective pre-post research conducted at a tertiary care center in Nagpur, Hingna, India. The cases included were of moderate to severe or worse OSA, older than 18 years, willing for CPAP therapy with no previous or current CPAP therapy. They had a history of excessive drowsiness during the day. The cases excluded from the study were those with an active, persistent breathing ailment requiring treatment, intervention, or diagnosis of dyslipidemia, diabetes mellitus, or hypertension, past or present, or evidence of damage to the vital end organs. Components of the metabolic syndrome were assessed at the beginning and end of three months of CPAP therapy.

Findings

Eighty-five cases were enrolled in the study, of which 79 completed it. The majority of cases were male, comprising 48 individuals, accounting for 60.8% of the total cohort. Additionally, 54 cases, representing 68.4% of the group, had hypertension. The average age of the participants was 53.95 years ± 6.84 years. The BMI mean was 30.4 kg/m^2^ ± 4.642, with a waist-hip ratio of 0.964 ± 0.056 and a neck circumference of 40.66 cm ± 3.37. The study population scored 12.53 ± 2,616 on the Epworth Sleepiness Scale. The study population’s apnea-hypopnea index/respiratory disturbance index ratio was 16.118 ± 4.868, a moderate risk score. After three months of CPAP therapy, there was a significant improvement in glycated hemoglobin (HbA1c), tetraiodothyronine (T4), high-density lipoprotein (HDL), and oxygen desaturation, and they were also statistically significant. In the study group, there was a decrease in systolic and diastolic blood pressure of 2.21 mm Hg and 0.26 mm Hg, respectively. Other indicators, including HbA1c, fasting and post-meal blood sugar, triglycerides, and HDL cholesterol, were significantly lower. We observed in the less than 50-year-old age group better improvement in systolic blood pressure of 0.49 mm Hg, diastolic blood pressure improvement of 0.32 mm Hg, and fasting blood sugar improvement of 14.59 mg/dl, and in the age group of more than 50, better improvements in post-meal blood sugar of 9.7 mg/dl, along with a statistically significant change in triglyceride with an improvement of 16.26 mg/dl, P value less than 0.05.

Interpretation

After three months of CPAP therapy, there was a significant improvement in HbA1c, T4, HDL, and oxygen desaturation, and they were also statistically significant. Fourteen (17.72%) cases of post-CPAP therapy no longer met the requirements for the syndrome. There was an improvement in the blood pressure’s diastolic and systolic values, fasting and post-prandial sugar levels, HbA1C, and triglyceride levels. Patients over 50 years old showed better improvement in post-meal and triglyceride levels. Females improved blood pressure and triglycerides, whereas males responded better to blood sugar levels.

## Introduction

Two-thirds of all fatalities globally each year are caused by noncommunicable diseases, primarily chronic respiratory diseases, cancer, and cardiovascular disorders [1]. A high frequency of metabolic syndrome and its comorbidities is linked with obstructive sleep apnea (OSA). The three essential elements of the metabolic syndrome are improper lipid metabolism, hypertension, and insulin resistance. The risk of cardiovascular disease is increased by both OSA and metabolic syndromes [2]. For symptomatic OSA, nonstop continuous positive airway pressure (CPAP) is the preferred therapeutic intervention. The study aimed to evaluate how CPAP therapy affects the metabolic syndrome and related symptoms, which included blood sugar level as indirect evidence of insulin sensitivity, obesity indices, which were calculated by measuring neck circumference, abdominal girth, waist-hip ratio, lipid abnormalities on a blood test, alertness through questionnaires and OSA, and a history of hypertension.

## Materials and methods

This current study is a prospective pre-post research. The study was conducted at a tertiary treatment center in Nagpur, Hingna, India. We recruited 85 cases; however, only 79 cases completed the study. The patients attending the outpatient department at the respiratory medicine department who were willing to participate included patients with moderate to severe or worse OSA, older than 18 years, willing for CPAP therapy with no previous or current CPAP therapy, and a history of excessive daytime sleepiness. The cases excluded from the study were those with an active, persistent obstructive airway disease requiring inhaled bronchodilators, interventions, or evidence of damage to the vital organs. Patient confidentiality was preserved, written informed consent was obtained from the patient, and the Institutional Ethics Committee (IEC) of Narendra Kumar Prasadrao Salve Institute of Medical Sciences & Research Centre and Lata Mangeshkar Hospital, Nagpur, approved the study (approval number IEC/NKPSIMS/3/2019).

The study aimed to evaluate how CPAP therapy affects the metabolic syndrome and related symptoms, which included blood sugar level as indirect evidence of insulin sensitivity, obesity indices, which were calculated by measuring neck circumference, abdominal girth, waist-hip ratio, lipid abnormalities on routine blood testing, assessment of alertness through questionnaires and OSA, and a history of hypertension. The utility of CPAP therapy in adults suffering from OSA with metabolic syndrome was noted in the above parameters.

Hypertension, stroke, and cardiovascular illness have all been linked to OSA syndrome. Chronic intermittent hypoxia, which deranges the lipid and glucose metabolism, is a feature of OSA. Metabolic syndrome, also called insulin resistance syndrome, is recognized as a constellation of obesity, glucose intolerance, dyslipidemia, and hypertension. The National Cholesterol Education Program Adult Treatment Panel III scale was used to determine metabolic syndrome [3]. At least three of the subsequent requirements have to be present for a metabolic syndrome diagnosis: blood pressure greater than 130/85 mm Hg, fasting blood glucose levels more significant than 110 mg/dl, waist circumference greater than 102 cm for men or higher than 88 cm for women, plasma triglyceride >150 mg/dl, and high-density lipoprotein (HDL) cholesterol <40 mg/dl in men or <50 mg/dl in women. A total of three or more was regarded as indicative of the occurrence of metabolic syndrome. Scores were given for each criterion, and the total was calculated. The metabolic syndrome elements were assessed at the beginning and end of three months of CPAP therapy. We evaluated the polysomnography parameters, anthropometric variables, blood pressure, and laboratory tests.

The initial assessment was done with an Epworth Sleepiness Scale (ESS) questionnaire and a simple evaluation model that consists of eight questions and includes the following: a history of snoring (S), fatigue/tiredness (T) throughout the day, breathing stopping observed while sleeping (O), high blood pressure (P), BMI (B) more significant than 30 kg/m^2^, age (A) greater than 50 years, neck circumference (N) greater than 40 cm, and male gender (G) that begin with the acronym STOP-BANG. It is graded based on yes-or-no responses. As a result, the scores range from zero to eight, and more than three suggest OSA. OSA syndrome is an amalgamation of excessive daytime sleepiness and OSA. The ESS was utilized to quantify excessive daytime sleepiness, with a score of 10 or more indicating extreme drowsiness during the day [4]. Each participant underwent an overnight polysomnography, and the outcomes were manually assessed following the American Academy of Sleep Medicine recommendations [5,6]. OSA is an apnea-hypopnea index (AHI) of five or more per hour of sleep. The severity was categorized as mild for a score of five to less than 15 and moderate for a score of 15 to 30. A score of over 30 on the AHI has been deemed severe OSA. The effectiveness of patients’ sleep was measured. Sleep efficiency is the percentage of time spent sleeping in a bed. It is calculated by dividing the total time spent in bed by the amount spent sleeping (in minutes). For a seven- to eight-hour sleep session, a sleep efficiency of 80% or higher is considered within the healthy range [7].

The anthropometric items studied were body weight and height. Obesity indices for the study were BMI, waist-hip ratio, and neck circumference. The threshold values for the BMI were for underweight: 18.5 kg/m^2^, healthy: 18.5-24.9 kg/m^2^, overweight: 25-29.9 kg/m^2^, and obese: 30 kg/m^2^ and beyond, whereas for waist-hip ratio, as stated by the World Health Organization, the normal range is 0.80 or less for women and 0.95 or less for men [7]. The neck circumference was measured, and the average values for adults were 14-19 inches (35.5-48.3 cm), or on average, 15 inches [8]. OSA risk predictability is higher for men with a neck circumference of 17 inches or more (43.18 cm), and for females, it is 16 inches or more (40.64 cm). After a minimum of five minutes of rest, blood pressure was assessed on patients sitting using a mercury sphygmomanometer that was periodically calibrated. The average of the three observed measurements was recorded. Hypertension was defined as blood pressure greater than 130/85 mm Hg [9,10].

In laboratory tests, we estimated the fasting blood sugar, post-meal blood sugar, glycated hemoglobin (HbA1C), and thyroid profile. The lipid profile components measured were triglycerides and HDL. Blood testing parameters included hemoglobin and total leucocyte count (TLC). The study population’s average blood sugar levels were as follows: fasting blood sugar levels span from 70 to 110 mg/dl, postprandial blood sugar levels two hours after a meal range from 70 to 140 mg/dl, random blood sugar levels span from 70 to 140 mg/dl, and prediabetes extent from 101 to 125 mg/dl, 141 to 200 mg/dl, and 140 to 200 mg/dl, respectively, for fasting, post-meal, random blood sugar, and for diabetes levels above it [11]. The usual range of HbA1c in adults is 4-5.6%. An elevated risk of developing diabetes and prediabetes is indicated by HbA1c values ranging from 5.7 to 6.4% and diabetes of 6.5% or more [12]. The thyroid profile average values for the study were thyroid-stimulating hormone (TSH) 0.5-4.5 mlU/l, tetraiodothyronine (T4) 5.4-11.5 mcg/dl, and triiodothyronine (T3) 60-180 ng/dl [13]. The lipid profile variables in the normal range were cholesterol less than 200 mg/dl, triglyceride less than 150 g/dl, HDL cholesterol more than 60 mg/dl, a borderline risk range of 200-239 mg/dl, 150-199 mg/dl, 35-45 mg/dl, high-risk range of 240 mg/dl, 200-499 mg/dl, and 160-189 mg/dl, respectively [14]. We also estimated hemoglobin, for which the normal range for men was 13.5-17.5 g/dl, while for women, it was 12-15.5 g/dl. The effect on TLC was observed, typically ranging from 4.5 to 12 × 103 cells per cubic millimeter of blood.

The CPAP machine usage was taught to the patient by a trained technician, and compliance was monitored regularly every seven days by the data recruited from the secure digital memory card.

The statistical program Epi Info^TM^ (CDC, Atlanta, Georgia, United States) was used for the statistical analysis. Summaries of continuous data were expressed as means, SDs, or medians (with IQRs), whereas summaries of categorical variables were expressed as proportions. A paired t-test was employed to examine the effects of CPAP therapy before and after, assuming that the data were normally distributed. P values on both sides were considered statistically significant if they were less than 0.05.

## Results

Baseline peculiarities of the study population

**Figure 1:**
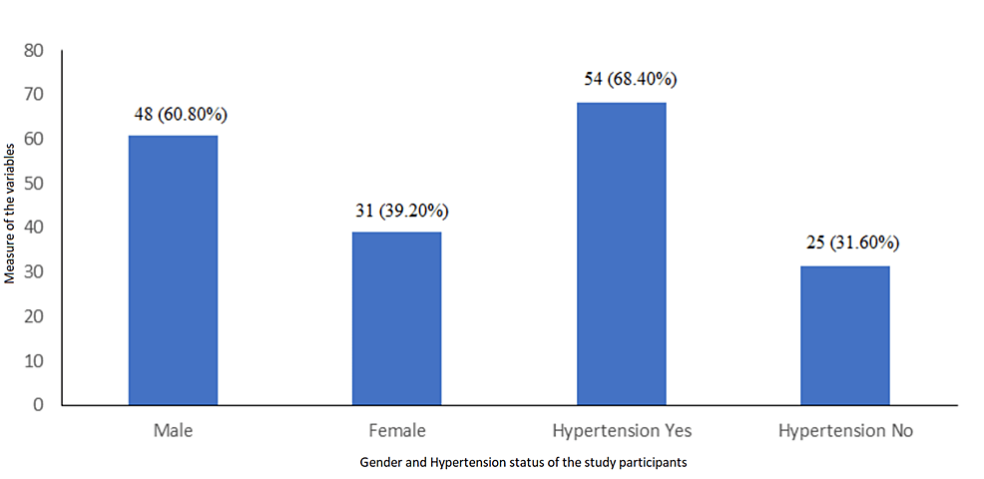
Baseline characteristics of the study population (N = 79 (100%))

Eighty-five cases were enrolled, of whom 79 completed the study. Most cases were males, 48 (60.8%), and 54 (68.4%) had hypertension, as shown in Figure 1. The mean age of the study population was 53.95 years ± 6.84.

It was observed in the obesity indices that the mean BMI was 30.4 kg/m^2^ ± 4.642, a waist-hip ratio of 0.964 ± 0.056, and a neck circumference of 40.66 cm ± 3.37, suggestive of obesity (Table 1).

**Table 1:** Baseline obesity indices and effect of CPAP therapy after three months of intervention (N = 79) P value is significant if less than 0.05. CPAP, continuous positive airway pressure

Obesity indices			After three months of CPAP therapy		P value
Variables	Mean	SD	Mean	SD	
BMI in kg/m^2^	30.4	4.642	31.5	4.34	0.0286
Waist-hip ratio	0.964	0.056	0.969	0.042	0.0581
Neck circumference	40.66	3.37	39.21	2.21	0.0462

The study population STOP-BANG was 3.25 ± 1.044, which was suggestive of being positive for OSA, and a score of 12.53 ± 2.616 on ESS indicated excessive daytime sleepiness. The AHI and respiratory disturbance index of the study population were 16.118 ± 4.868, with a score of moderate OSA (Table 2).

**Table 2:** Baseline results of polysomnography variables (N = 79) STOP-BANG: a history of snoring (S), fatigue/tiredness (T) throughout the day, breathing stopping observed while sleeping (O), high blood pressure (P), BMI (B) greater than 30 kg/m^2^, age (A) greater than 50 years, neck circumference (N) greater than 40 cm, and male gender (G) AHI, apnea-hypopnea index; ESS, Epworth Sleepiness Scale; RDI, respiratory disturbance index

Variable	Mean	SD
STOP-BANG questionnaire	3.25	1.044
ESS	12.53	2.616
Apnea	45.15	14.774
Hypopnea	50.24	17.476
Total sleep time in hours	6.021	0.656
AHI	16.118	4.868
RDI	16.118	4.868

There was a favorable improvement in HbA1c, TLC, T4, TSH, HDL, and oxygen desaturation, and they were also statistically significant (P value < 0.05), with slight or no significant changes observed in other variables (Table 3).

**Table 3:** Baseline results of laboratory variables with the effect of CPAP therapy after three months of intervention (N = 79) P value is significant if less than 0.05. CPAP, continuous positive airway pressure; Hb, hemoglobin; HbA1c, glycated hemoglobin; HDL, high-density lipoprotein; T3, triiodothyronine; T4, tetraiodothyronine; TLC, total leucocyte count; TSH, thyroid-stimulating hormone

Baseline laboratory values			After three months of CPAP therapy		P value
Variable	Mean	SD	Mean	SD	
Hb in gm/dl	12.818	1.458	12.512	1.408	0.0461
HbA1c in g %	7.797	0.939	6.3	0.125	<0.001
TLC/cumm	7,185.75	1,862.626	7,110	1,688.61	0.0454
T3 in ng/ml	2.059	1.022	2.158	2.125	0.0816
T4 in ug/dl	2.076	0.9	4.05	0.81	<0.001
TSH ulU/ml	2.818	3.394	2.918	4.152	0.0456
HDL in mg/dl	43.61	9.163	48	5.12	<0.001
Oxygen desaturation	80.46	7.209	90.41	5.421	<0.001

Impact of intervention on metabolic syndrome outcomes

**Figure 2:**
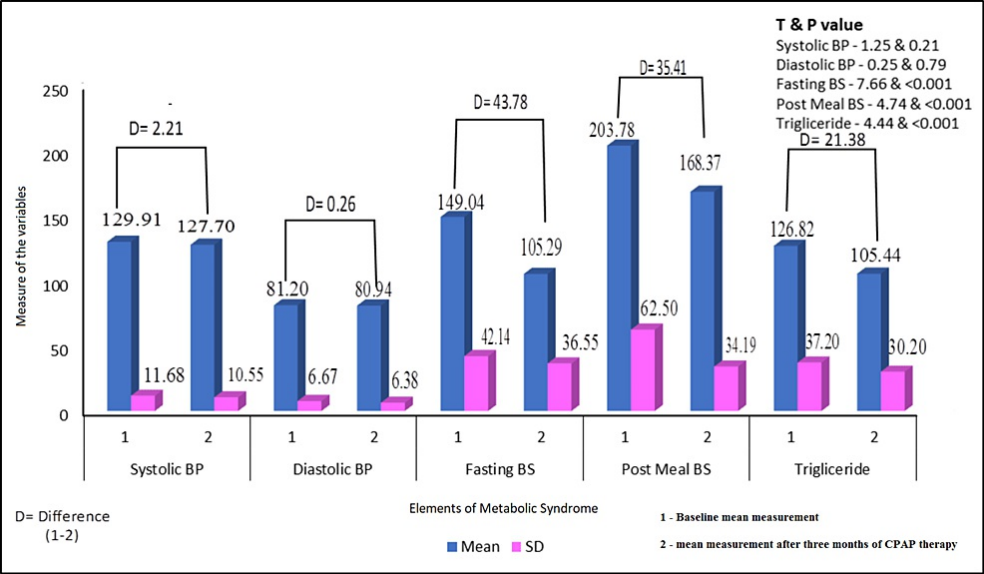
Effects of CPAP therapy on metabolic syndrome elements (N = 79) P value is significant if less than 0.05. BP, blood pressure; BS, blood sugar; CPAP, continuous positive airway pressure; D, difference

Figure 2 shows the effect of CPAP therapy after three months of intervention on the elements of metabolic syndrome. The elements of the metabolic syndrome baseline measurement were compared to the effect of the CPAP therapy after three months. The difference (D) between the baseline and change after three months of intervention was estimated. At the beginning of their CPAP therapy, 79 cases had metabolic syndrome. Following CPAP therapy, the frequency of the metabolic syndrome decreased (Table 3; Figure 2). Fourteen (17.72%) of the 79 cases with metabolic syndrome who went through CPAP therapy no longer met the criteria for the syndrome. Before using CPAP therapy, the metabolic syndrome score was 3.95 ± 0.56; after using CPAP, it was 2.74 ± 0.95, with a P value equal to 0.001. The specific yardsticks that resolved in the 14 cases with metabolic syndrome following CPAP therapy included four each in fasting blood glucose, triglyceride levels, and HDL cholesterol, whereas two patients had normal blood pressure. None showed improvement in abdominal circumference.

There was a significant improvement in fasting and post-meal blood sugar and triglyceride levels, which were also statistically significant, with a slight improvement in blood pressure indices, of which the systolic blood pressure improvement was statistically significant.

Outcomes based on the results of metabolic syndrome elements and anthropometric variables

Following three months of CPAP therapy, obesity indices demonstrated a slight increase in BMI (31.5 kg/m^2^ ± 4.34) being statistically significant, but no appreciable change in neck circumference (0.969 ± 0.042) or waist-hip ratio (39.21 ± 2.21) (Table 1). After CPAP therapy, there was a decrease in systolic and diastolic blood pressure of 2.21 mm Hg and 0.26 mm Hg, respectively. Other indicators, including HbA1c, triglycerides, and HDL cholesterol, were significantly lower by 1.49%, 21.38 mg/dl, and 4.39 mg/dl, which was also statistically significant with a P value less than 0.05 (Table 3).

Serious and unfavorable adverse events

Accelerated hypertension developed in one patient while receiving CPAP therapy, and antihypertensive medication was given to this patient. Due to CPAP therapy sensitivity, two patients stopped using it after the first month. Other adverse effects included skin irritation in 51% of cases, discomfort in the nasal bridge in 44% of cases, nasal congestion in 28% of cases, headaches in 26% of cases, and mask leaks in 30% of cases.

Outcomes on elements of the metabolic syndrome correlated with age and gender

**Figure 3:**
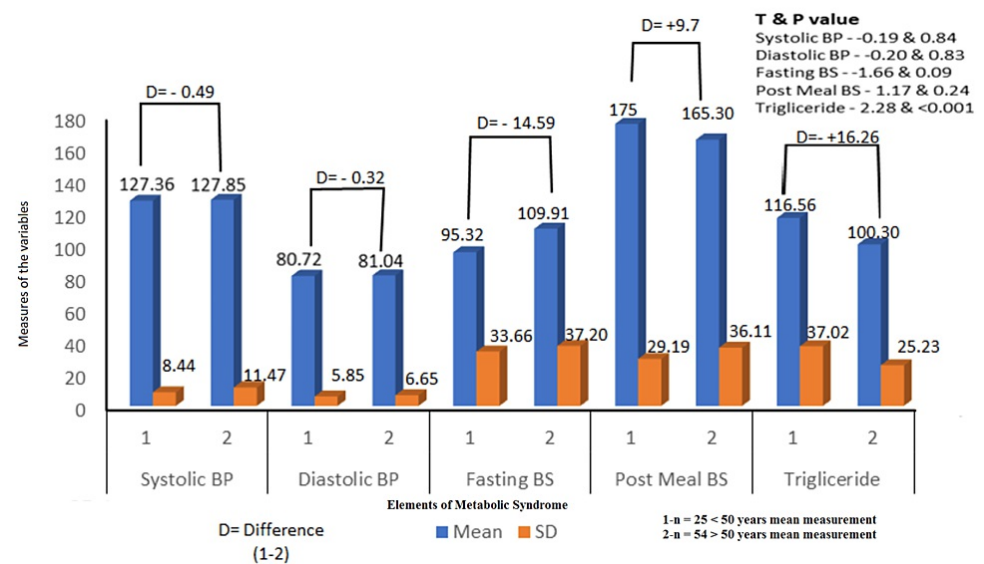
CPAP therapy effects on metabolic syndrome elements in the age group below and above 50 years (N = 79) P value is significant if less than 0.05. 1-n, sample size of age group less than 50 years with their mean measure; 2-n, sample size of age group more than 50 years with their mean measure; BP, blood pressure; BS, blood sugar; CPAP, continuous positive airway pressure; D, difference

We classified the study population as above 50 with a sample size of 25 and below 50 years old, a sample size of 54, and studied the effect of three months of CPAP therapy on the elements of metabolic components, as shown in Figure 3. The difference (D) between them was estimated. We observed that the less than 50-year-old age group showed better improvement in systolic blood pressure of 0.49 mm Hg, diastolic blood pressure improvement of 0.32 mm Hg, fasting blood sugar improvement of 14.59 mg/dl, and the age group of more than 50 showed better improvements in post-meal blood sugar of 9.7 mg/dl, along with a statistically significant change in triglyceride with an improvement of 16.26 mg/dl, P value less than 0.001.

**Figure 4:**
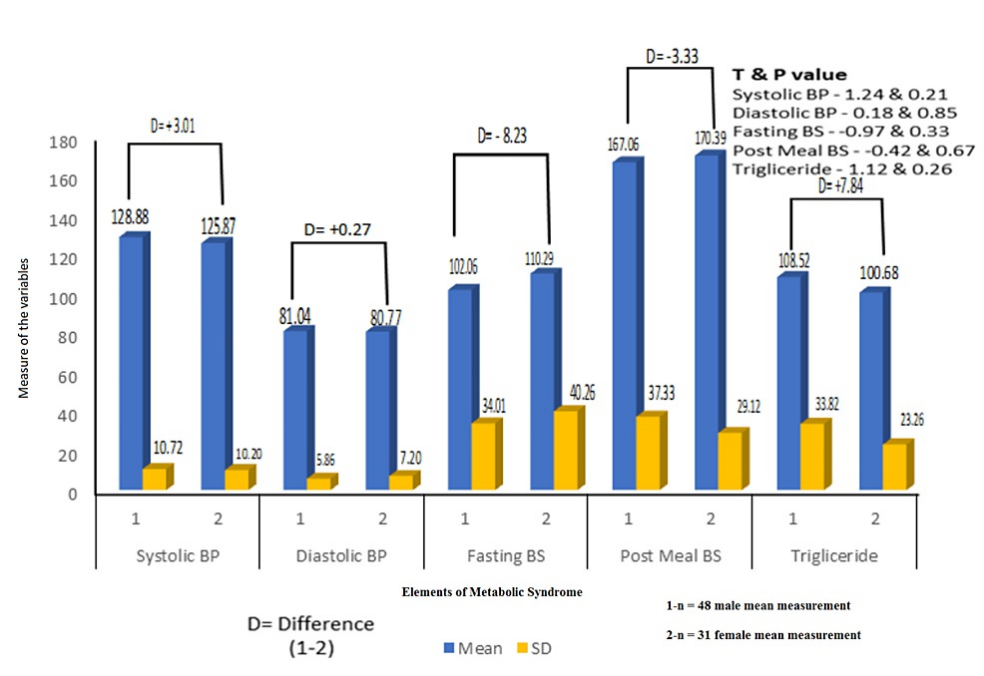
CPAP therapy effects on metabolic syndrome elements in males and females (N = 79) P value is significant if less than 0.05. 1-n, sample size of male patients with their mean measure; 2-n, sample size of female patients with their mean measure; BP, blood pressure; BS, blood sugar; CPAP, continuous positive airway pressure; D, difference

We also studied the effect of CPAP therapy on elements of metabolic syndrome in males with a sample size of 48 and females with a sample size of 31, as shown in Figure 4. The difference (D) of the mean of each variable was estimated. We observed females did better in blood pressure, with an improvement of 3.01 mm Hg in systolic blood pressure and 0.27 mm Hg in diastolic blood pressure, and in triglyceride level, with a better chance of 7.84 mg/dl. In contrast, males had a better response to blood sugar levels, with fasting blood sugar improvement of 8.23 mg/dl and post-meal blood sugar improvement of 3.33 mg/dl, with statistically significant changes in systolic blood pressure, fasting blood sugar, and triglyceride levels.

## Discussion

OSA, along with metabolic syndrome, synergizes the risk of various systemic disorders, with incremental effects on morbidity and mortality. CPAP is the preferred treatment for diagnosed OSA. This study population was selected because it had a higher risk of complications and death. To lower the intensity of confounding effects, patients with OSA who had not earlier received CPAP therapy were included in the trial.

Eighty-five cases that met the inclusion and exclusion criteria were incorporated into the study, of which 79 completed the study. The larger part of the study population was male, 48 (60.8%), with a mean age of 53.95 years, and 54 (68.4%) of cases had hypertension. The ESS and STOP-BANG scores were 12.53 ± 2.616 and 3.25 ± 1.044, respectively, suggestive of daytime sleepiness. The AHI index was 16.118 ± 4.868, indicating moderate OSA. The total sleep time in hours was 6.021 ± 0.656.

The major obesity indices studied were BMI, waist-hip ratio, and neck circumference. Our study found a slight increase in BMI of 31.5 ± 4.34 kg/m^2^ with no appreciable change in the other two variables after three months of CPAP therapy. Hoffstein and Mateika, in a study of 670 patients, Ahbab et al., and Kim et al. observed an increase in all the above indices and also determined them as an independent risk factor for OSA [15-17]. Redenius et al., Garcia et al., Drager et al., Ng et al., and Ou et al. observed that the BMI and weight either increased or there was no change after CPAP therapy [18-22]. However, Pleava et al. found a significant decline in BMI with a more considerable decrease in abdominal circumference following CPAP therapy [23].

In the laboratory parameters, we assessed the HbA1C, and a favorable change of 1.497% was noted. We compared our findings with a few previous studies, as shown in Table 4.

**Table 4:** A summary of similar studies along with their references CPAP, continuous positive airway pressure; HbA1C, glycated hemoglobin; HOMA-IR, Homeostatic Model Assessment for Insulin Resistance

Study	Sample size	Design/outcome measurements	Primary finding	Year
Davies et al. [24]	10	Cohort-matched control fasting insulin and lipid profile for three months	No change in insulin level with CPAP	1994
Czupryniak et al. [25]	9	Continuous glucose monitoring, plasma insulin, and HOMA-IR	Mean blood glucose, fasting insulin, and HOMA-IR were significantly higher with CPAP treatment	2005
Prasad et al. [26]	221	Retrospective cohort; CPAP effects over two years	No change in HbA1C before and after CPAP treatment	2012
Guest et al. [27]	300	Retrospective case-control; CPAP vs. no therapy over five years	HbA1C is significantly lower in the CPAP group	2014
Guo et al. [28]	40	Prospective: pre-treatment vs. post-CPAP therapy for one month	Decreased 24-hour mean blood glucose and nighttime mean blood glucose after CPAP, as determined by continuous glucose monitoring	2015
Our study	79	Cross-sectional observational	HbA1C, fasting, and post-meal blood sugar showed significant improvement	2022

In our study, we observed significant improvements in HDL and triglycerides. Nadeem et al., Shehab et al., Pazarli et al., and Asgari et al., similar to our study, observed that post-CPAP therapy decreases triglycerides and total cholesterol with an increase in HDL; however, on the contrary, Xu et al. and Chen et al. suggested no clinically significant effect on lipid metabolism [29-34].

In our study, there was only a slight improvement in blood pressure parameters after three months of CPAP therapy; however, Asgari et al. and Haentjens et al. observed a significant reduction in blood pressure post-CPAP therapy [32,35].

Our study showed significant improvement in T4, with a slight improvement in T3, TSH, and TLC. Bahammam et al. observed that OSA patients had more subclinical hypothyroidism [36]. Sökücü et al. observed that post-six-month CPAP therapy improves mean platelet distribution, red cell distribution width, and platelet volume [37]. However, Bielicki et al. found no difference in thyroid disorders between OSA and the general population [38].

We also studied the effect of CPAP on the age groups above and below 50 years and in the male and female populations. We observed a less than 50-year-old age group showing better improvement in systolic blood pressure of 0.49 mm Hg, diastolic blood pressure improvement of 0.32 mm Hg, fasting blood sugar improvement of 14.59 mg/dl, and an age group of more than 50 showing better improvements in post-meal blood sugar of 9.7 mg/dl and triglyceride with an improvement of 16.26 mg, both statistically significant, P value less than 0.05. However, when compared with gender, females did better in blood pressure with an improvement of 3.01 mm Hg in systolic blood pressure and 0.27 mm Hg in diastolic blood pressure, and in triglyceride level with a better change of 7.84 mg/dl. In contrast, males responded better to blood sugar levels with a fasting blood sugar improvement of 8.23 mg/dl and a post-meal blood sugar improvement of 3.33 mg/dl, though none were statistically significant.

The limitation of this study being observational is that a randomized control trial with a comparative group would be more helpful.

## Conclusions

The study showed improvement in laboratory parameters, with a slight or no change in obesity indices. Few of the patients post-CPAP therapy no longer fulfilled the syndrome’s requirements. There was an improvement in both systolic and diastolic blood pressure in patients less than 50 years of age and females. This study shows that CPAP therapy can improve the patient clinically and help reverse metabolic syndrome. However, it will take more investigation to pinpoint the precise mechanisms at play.

## References

[1] The Lancet (2011). Time for action in New York on non-communicable diseases. Lancet.

[2] Lévy P, Bonsignore MR, Eckel J (2009). Sleep, sleep-disordered breathing and metabolic consequences. Eur Respir J.

[3] (2001). Executive summary of the third report of the National Cholesterol Education Program (NCEP) Expert Panel on Detection, Evaluation, and Treatment of High Blood Cholesterol in Adults (Adult Treatment Panel III). JAMA.

[4] Johns MW (1991). A new method for measuring daytime sleepiness: the Epworth sleepiness scale. Sleep.

[5] Sharma SK, Kurian S, Malik V (2004). A stepped approach for prediction of obstructive sleep apnea in overtly asymptomatic obese subjects: a hospital based study. Sleep Med.

[6] Epstein LJ, Kristo D, Strollo PJ (2009). Clinical guideline for the evaluation, management and long-term care of obstructive sleep apnea in adults. J Clin Sleep Med.

[7] Nishida C (2004). Appropriate body-mass index for Asian populations and its implications for policy and intervention strategies. Lancet.

[8] Davies RJ, Stradling JR (1990). The relationship between neck circumference, radiographic pharyngeal anatomy, and the obstructive sleep apnoea syndrome. Eur Respir J.

[9] Chopra HK, Ram CV (2019). Recent guidelines for hypertension: a clarion call for blood pressure control in India. Circ Res.

[10] Wright JD, Hughes JP, Ostchega Y, Yoon SS, Nwankwo T (2011). Mean systolic and diastolic blood pressure in adults aged 18 and over in the United States, 2001-2008. Natl Health Stat Report.

[11] (2023). Diabetes. https://www.mayoclinic.org/diseases-conditions/diabetes/symptoms-causes/syc-20371444.

[12] (2023). All about your A1C. https://www.cdc.gov/diabetes/managing/managing-blood-sugar/a1c.html.

[13] (2023). Thyroid. https://www.healthcentral.com/search?q=Thyroid.

[14] (2023). Lipid profile. https://www.hopkinsmedicine.org/search?form_instance=enterprise&q=lipid+profile.

[15] Hoffstein V, Mateika S (1992). Differences in abdominal and neck circumferences in patients with and without obstructive sleep apnoea. Eur Respir J.

[16] Ahbab S, Ataoğlu HE, Tuna M, Karasulu L, Cetin F, Temiz LU, Yenigün M (2013). Neck circumference, metabolic syndrome and obstructive sleep apnea syndrome; evaluation of possible linkage. Med Sci Monit.

[17] Kim SE, Park BS, Park SH, Shin KJ, Ha SY, Park J (2015). Predictors for presence and severity of obstructive sleep apnea in snoring patients: significance of neck circumference. J Sleep Med.

[18] Redenius R, Murphy C, O’Neill E, Hamwi MA, Zallek SN (2008). Does CPAP lead to change in BMI?. J Clin Sleep Med.

[19] Garcia JM, Sharafkhaneh H, Hirshkowitz M, Elkhatib R, Sharafkhaneh A (2011). Weight and metabolic effects of CPAP in obstructive sleep apnea patients with obesity. Respir Res.

[20] Drager LF, Brunoni AR, Jenner R, Lorenzi FG, Bensenor IM, Lotufo PA (2015). Effects of CPAP on body weight in patients with obstructive sleep apnoea: a meta-analysis of randomised trials. Thorax.

[21] Ng SS, Liu EK, Ma RC (2017). Effects of CPAP therapy on visceral fat thickness, carotid intima-media thickness and adipokines in patients with obstructive sleep apnoea. Respirology.

[22] Ou Q, Chen B, Loffler KA (2019). The effects of long-term CPAP on weight change in patients with comorbid OSA and cardiovascular disease: data from the SAVE Trial. Chest.

[23] Pleava R, Mihaicuta S, Serban CL, Ardelean C, Marincu I, Gaita D, Frent S (2020). Long-term effects of continuous positive airway pressure (CPAP) therapy on obesity and cardiovascular comorbidities in patients with obstructive sleep apnea and resistant hypertension-an observational study. J Clin Med.

[24] Davies RJ, Turner R, Crosby J, Stradling JR (1994). Plasma insulin and lipid levels in untreated obstructive sleep apnoea and snoring; their comparison with matched controls and response to treatment. J Sleep Res.

[25] Czupryniak L, Loba J, Pawlowski M, Nowak D, Bialasiewicz P (2005). Treatment with continuous positive airway pressure may affect blood glucose levels in nondiabetic patients with obstructive sleep apnea syndrome. Sleep.

[26] Prasad B, Carley DW, Krishnan JA, Weaver TE, Weaver FM (2012). Effects of positive airway pressure treatment on clinical measures of hypertension and type 2 diabetes. J Clin Sleep Med.

[27] Guest JF, Panca M, Sladkevicius E, Taheri S, Stradling J (2014). Clinical outcomes and cost-effectiveness of continuous positive airway pressure to manage obstructive sleep apnea in patients with type 2 diabetes in the U.K. Diabetes Care.

[28] Guo LX, Zhao X, Pan Q (2015). Effect of continuous positive airway pressure therapy on glycemic excursions and insulin sensitivity in patients with obstructive sleep apnea-hypopnea syndrome and type 2 diabetes. Chin Med J (Engl).

[29] Nadeem R, Singh M, Nida M (2014). Effect of CPAP treatment for obstructive sleep apnea hypopnea syndrome on lipid profile: a meta-regression analysis. J Clin Sleep Med.

[30] Shehab WA, Elhabashy M (2019). The effect of continuous positive airway pressure on dyslipidemia in patients with obstructive sleep apnea. Egypt J Bronchol.

[31] Pazarli A, Koseoglu H, Kanbay A, Abakay M (2018). The effect of positive airway pressure therapy on lipid profile. Eurasian J Pulmonol.

[32] Asgari A, Soltaninejad F, Farajzadegan Z, Amra B (2019). Effect of CPAP therapy on serum lipids and blood pressure in patients with obstructive sleep apnea syndrome. Tanaffos.

[33] Xu H, Yi H, Guan J, Yin S (2014). Effect of continuous positive airway pressure on lipid profile in patients with obstructive sleep apnea syndrome: a meta-analysis of randomized controlled trials. Atherosclerosis.

[34] Chen B, Guo M, Peker Y (2022). Effect of continuous positive airway pressure on lipid profiles in obstructive sleep apnea: a meta-analysis. J Clin Med.

[35] Haentjens P, Van Meerhaeghe A, Moscariello A, De Weerdt S, Poppe K, Dupont A, Velkeniers B (2007). The impact of continuous positive airway pressure on blood pressure in patients with obstructive sleep apnea syndrome: evidence from a meta-analysis of placebo-controlled randomized trials. Arch Intern Med.

[36] Bahammam SA, Sharif MM, Jammah AA, Bahammam AS (2011). Prevalence of thyroid disease in patients with obstructive sleep apnea. Respir Med.

[37] Sökücü SN, Ozdemir C, Dalar L, Karasulu L, Aydın S, Altın S (2014). Complete blood count alterations after six months of continuous positive airway pressure treatment in patients with severe obstructive sleep apnea. J Clin Sleep Med.

[38] Bielicki P, Przybyłowski T, Kumor M, Barnaś M, Wiercioch M, Chazan R (2015). Thyroid hormone levels and TSH activity in patients with obstructive sleep apnea syndrome. Advances in Clinical Science.

